# Interprofessional Collaboration in Primary Healthcare: A Qualitative Study of General Practitioners’ and Family and Community Nurses’ Perspectives in Italy

**DOI:** 10.3390/healthcare13212794

**Published:** 2025-11-04

**Authors:** Federica Dellafiore, Luca Guardamagna, Sihame Haoufadi, Alice Cicognani, Angela De Mola, Benedetta Mazzone, Giulia Occhini, Antonio Brusini, Giovanna Artioli

**Affiliations:** 1 Department of Life Health Sciences and Health Professions, Link Campus University, 44, 00165 Roma, Italy; 2 Department of Orthopedics and Traumatology, Istituti Clinici di Pavia e Vigevano S.p.A., 27100 Pavia, Italy; 3 Laboratory for Rehabilitation and Orthopedic Surgery (LAROS), Institute of Care “Città di Pavia”, Department of Clinical Surgical, Diagnostic and Pediatric Sciences, University of Pavia, 27100 Pavia, Italy; 4 Community Health Centre of Russi, Local Health Unit (AUSL) Romagna, 48121 Ravenna, Italy; 5 Vascular Surgery and Internal Medicine, Local Health Unit (ASST) Crema, 26013 Crema, Italy; 6 Local Health Authority of Piacenza, Piazzale Milano 3, 29121 Piacenza, Italy; 7 Local Health Authority (ASL) Toscana Centro, Piazza Santé Maria Nuova 1, 50122 Florence, Italy; 8 Sports Medicine, Local Health Unit (AUSL) of Modena, 41125 Modena, Italy; 9 Department of Medicine and Surgery, University of Parma, 43125 Parma, Italy

**Keywords:** primary healthcare, chronic illness, health promotion, interprofessional collaboration, general practitioners, family and community nurses

## Abstract

**Background:** The growing burden of chronic illnesses calls for integrated and sustainable models of Primary Healthcare (PHC) that emphasize health promotion and patient-centered care. Interprofessional collaboration between General Practitioners (GPs) and Family and Community Nurses (FCNs) is a strategic approach to enhancing continuity of care and supporting individuals in adopting healthy behaviors across the trajectory of chronic conditions. This study aims to explore the experiences and perspectives of GPs and FCNs in Italy, with the goal of identifying the barriers, enablers, and transformative dynamics that can inform future PHC models. **Methods:** A qualitative study was conducted with four focus groups with 21 participants (8 GPs and 13 FCNs) from three Italian regions, carried out between March and November 2023. Data were analyzed using Reflexive Thematic Analysis (RTA) following Braun and Clarke’s framework. Ethical approval was obtained from the University of Parma (Protocol No. 0266537—21 October 2022). **Results:** Four themes and sixteen subthemes were identified: (1) barriers to effective collaboration (role ambiguity, limited time, structural misalignments); (2) facilitators of collaboration (openness, mutual recognition, shared goals); (3) team-building processes (phases of trust development, shared values, reflective problem-solving); and (4) transformation of work practices (improved patient outcomes, flexible methodologies, integrated care strategies). **Conclusions:** Interprofessional collaboration between GPs and FCNs enhances the capacity of PHC to address the complex needs of people with chronic conditions. Aligning relational, organizational, and structural factors is essential for sustainable, health-promoting care models. Tailored training, protected time, and shared spaces are critical to foster teamwork, promote patient empowerment, and ensure continuity of care in chronic illness management.

## 1. Background

The increasing complexity of chronic disease management, population aging, and health disparities has underscored the need to reconfigure healthcare systems toward models that emphasize continuity, prevention, and health promotion as central strategies for advancing population well-being and global health [1]. In this perspective, Primary Healthcare (PHC) is increasingly recognized not only as a setting for medical treatment, but also as a key arena for enabling healthier communities [2,3]. PHC, as initially articulated in the 1978 Declaration of Alma-Ata and reaffirmed in the 2018 Astana Declaration, is recognized as the foundation of an effective and equitable healthcare system. PHC prioritizes comprehensive, patient-centered care delivered through integrated, community-based services, aiming to improve access, outcomes, and system sustainability [4,5,6].

Within this framework, General Practitioners (GPs) and Family and Community Nurses (FCNs) are pivotal actors in translating the principles of PHC into everyday practice, bridging medical care, community engagement, and proactive approaches to health [5,6]. GPs traditionally act as gatekeepers to the health system and assume central responsibility for diagnosis, treatment, and referral [7,8,9], whereas FCNs increasingly embody a proactive, population-oriented model of care rooted in community engagement, chronic disease management, and health promotion [10,11]. Although both roles are critical to achieving the goals of PHC, evidence increasingly recognizes their collaboration as essential, with a growing body of literature underscoring the potential benefits of stronger GP–FCN partnerships [12,13]. Effective interprofessional relationships are associated with improved patient outcomes, greater continuity of care, and enhanced responsiveness to the needs of socially complex and multimorbid populations who particularly benefit from coordinated, multidisciplinary input [14,15]. At the professional level, such collaboration has also been linked to greater job satisfaction, stronger role identity, and reduced professional isolation among both GPs and FCNs [16]. These findings underscore the importance of interprofessional teamwork as a cornerstone of sustainable and patient-centered PHC delivery.

Despite these recognized benefits, the operationalization of GP–FCN collaboration remains challenging. To better understand these challenges, it is useful to refer to the broader concept of interprofessional work, defined as the process in which multiple health professionals from different backgrounds collaborate with patients, families, and communities to deliver the highest quality of care through shared decision-making and coordinated practice [17]. The analysis is informed by the framework proposed by Reeves et al. (2010), which conceptualizes interprofessional collaboration as a dynamic process based on shared goals, mutual respect, and coordinated practice [18]. In this model, collaboration develops through the interaction of relational factors—such as trust and communication—and organizational factors—such as shared structures and leadership support—that enable effective teamwork in PHC [18]. Interprofessional work models have been shown to enhance care delivery by improving coordination, continuity, and the patient-centeredness of services, leading to better health outcomes and greater professional satisfaction [18].

The empirical literature and professional accounts converge in identifying persistent obstacles to interprofessional collaboration, including unclear role delineation, tensions related to professional identity within traditional hierarchies, and fragmented organizational cultures that impede shared decision-making [15,19]. For instance, GPs and FCNs often report uncertainty about each other’s responsibilities and scope of practice, leading to duplication of tasks or missed opportunities for coordinated care. Communication is frequently informal and episodic rather than structured and continuous, which limits systematic care coordination and the development of reliable collaborative routines [20]. These barriers can result in underutilization of FCN expertise and hinder the full realization of integrated primary care benefits [15]. Conversely, enabling factors such as shared goals, mutual trust, and open, structured communication foster effective collaboration and support coordinated patient-centered care. Concrete examples include regular team meetings, joint care planning sessions, and the use of shared documentation tools, which help clarify roles, align expectations, and strengthen professional relationships. Overall, interprofessional collaboration is shaped by both relational and structural factors; acknowledging these dynamics is essential to strengthen GP–FCN collaboration within the Italian PHC context and to inform strategies for sustainable, high-quality primary care [14,15,16,20].

Although preliminary research has begun to shed light on these dynamics [21,22], the empirical evidence remains limited and fragmented. In particular, little is known about how collaboration is conceptualized and enacted in everyday primary care practice, and how the expectations and lived experiences of professionals shape these processes [23,24]. While previous studies have explored interprofessional collaboration internationally [22,25,26], there is a lack of in-depth qualitative evidence from the Italian PHC context, where organizational, cultural, and policy factors uniquely shape GP–FCN collaboration. By focusing on the Italian setting, this study provides novel insights into how interprofessional collaboration is experienced, operationalized, and influenced by national contextual factors, thus addressing a critical gap in the literature. Addressing this gap requires in-depth qualitative inquiry capable of capturing the complexity of interprofessional interactions and the contextual factors that enable or hinder them. Against this background, the present qualitative study seeks to explore how collaboration between GPs and FCNs is conceptualized, experienced, and operationalized within PHC. Accordingly, the study aims to explore the experiences and perspectives of GPs and FCNs in Italy, with the goal of identifying barriers, enablers, and transformative dynamics that can inform future PHC models. By examining the perceptions, expectations, and lived experiences of both professional groups, the study aims to generate empirically grounded insights that can inform the design of more integrated, patient-centered care models and guide future training and organizational strategies to support interprofessional teamwork in primary care.

## 2. Methods

### 2.1. Study Design

The study adopted a qualitative interpretative design with the aim of achieving an in-depth understanding of participants’ experiences. As outlined by Braun and Clarke (2019; 2021), data were generated through focus groups (FGs) and subsequently analyzed using Reflexive Thematic Analysis (RTA) [27,28]. RTA, grounded in a constructivist epistemological framework, emphasizes the identification and interpretation of patterns of meaning within qualitative data, while at the same time fostering the reflexive engagement of the researcher. Its methodological flexibility and focus on the co-construction of meaning render it particularly suitable for investigating complex professional and educational contexts, in contrast to other approaches such as phenomenology, grounded theory, or content analysis, which are primarily concerned with theory building or the exclusive analysis of lived experience.

In order to ensure methodological rigor and transparency, the study was conducted in accordance with the Reflexive Thematic Analysis Reporting Guidelines (RTARG) [29], which set out best practices and address common challenges in the reporting of RTA [30]. This approach facilitated the development of a rich and nuanced interpretative account of interprofessional collaboration in primary care, specifically focusing on the interactions between FNCs and GPs.

### 2.2. Setting and Participants

A purposive sampling strategy was employed to recruit healthcare professionals with substantial experience in structured interprofessional collaboration within primary healthcare settings. The approach aimed to identify information-rich cases capable of providing nuanced insights into the dynamics of collaborative practice. Eligible participants included GPs and FCNs actively employed in primary healthcare contexts, such as Community Health Centers, group medical practices, or territorial services. To ensure the relevance and richness of the data, participants were required to have at least one year of continuous, direct collaboration with the counterpart professional group, sufficient proficiency in Italian to engage meaningfully in FG discussions, and willingness to provide written informed consent. This criterion was adopted to ensure that participants had developed sufficient familiarity and experience within interprofessional contexts, allowing for them to provide in-depth, reflective accounts rather than initial or episodic perspectives. This requirement helped ensure that the data collected would reflect mature, informed perspectives on collaboration dynamics rather than preliminary or transient experiences.

Recruitment was carried out through institutional contacts and professional networks to capture a diverse range of perspectives across roles and practice settings. Participants were selected based on their potential contribution to the study aims, with emphasis on their capacity to provide detailed and reflective accounts of interprofessional collaboration. The study was conducted across three Italian regions—Tuscany, Lombardy, and Emilia-Romagna—which differ in population size and healthcare organization. Tuscany emphasizes community-based and integrated care models; Lombardy, Italy’s most populous region, features a mixed public–private system; and Emilia-Romagna is recognized for its strong territorial care networks and multidisciplinary collaboration. These contextual variations provided a meaningful background for exploring interprofessional practices across diverse healthcare settings. The final sample size was determined by data saturation, reached when additional FGs ceased to yield new insights or themes [28,31]. This approach ensured the inclusion of participants whose experiences were particularly informative for developing a comprehensive and contextually grounded understanding of interprofessional collaboration in primary care.

### 2.3. Data Collection and Instrument

Data were collected through four semi-structured FGs conducted between November 2022 and March 2023. Two FGs involved GPs and two involved FCNs, each lasting approximately 90 min. Specifically, FGs were conducted separately for GPs and FCNs to ensure participants could speak freely about their experiences without influence from hierarchical dynamics or role-related tensions, thus facilitating open and honest discussion. FGs were chosen as the method of data collection due to their ability to facilitate interaction among participants, elicit shared experiences, and capture collective dynamics, making them particularly suitable for exploring interprofessional collaboration in primary care [32,33]. Although no specific published framework was adopted, the focus groups were conducted following established qualitative guidelines for FG methodology [34] including structured questioning, iterative discussion, and reflexive facilitation to capture participants’ experiences and perspectives. Sessions were conducted either in-person or online via Microsoft Zoom, depending on participant availability. The format of each FGs (in-person or online) was determined based on participants’ geographic location and availability. Methodological consistency was ensured by applying the same facilitation techniques, discussion guide, and session structure across both formats. All sessions included detailed field notes taken by the moderator, which served the role of observer by capturing non-verbal cues, interactional dynamics, and contextual factors.

The focus group guide included open-ended prompts and probing questions specifically designed to explore participants’ experiences, perceptions, expectations, challenges, facilitators, and professional dynamics in interprofessional collaboration. The opening prompt for all sessions was: ‘How would you describe the relationship between GPs and FCNs?’ This broad question encouraged participants to freely construct and share their experiences. Subsequent discussion was guided by follow-up probes addressing communication practices, role clarity, teamwork strategies, and perceived barriers and enablers. Field notes were collected alongside the sessions to capture non-verbal cues, interactional dynamics, and contextual factors relevant to data interpretation.

All FGs were moderated by a senior researcher (PhD in Nursing Science; Associate Professor of Nursing Research) with extensive qualitative research experience, using an open-ended, conversational format designed to stimulate detailed narratives about collaboration experiences. The opening prompt for all sessions was: “*How would you describe the relationship between GPs and FCNs*?” This broad and open question allowed for participants to freely construct and share their experiences, without being constrained by narrowly defined questions. Subsequent discussion was guided by probing questions addressing perceptions, expectations, challenges, facilitators, and professional dynamics.

Moderators maintained a neutral and empathetic stance throughout, employing active listening techniques such as paraphrasing, summarizing, and interpretive questioning to encourage reflection, clarify meaning, and ensure accurate understanding of participants’ perspectives. A psychologically safe environment was fostered, encouraging participants to speak freely and providing sufficient space to articulate or reformulate their thoughts. Field notes were also collected to contextualize non-verbal cues, interactional dynamics, and other contextual factors relevant to data interpretation.

All sessions were audio-recorded with participants’ consent and transcribed verbatim. Audio recordings were transcribed verbatim using transcription software and subsequently checked manually for accuracy and completeness. Transcripts were anonymized, and participants were assigned numerical codes to ensure confidentiality. The integration of field notes with verbatim transcripts allowed for a more nuanced and contextually grounded understanding of the data. The full FG guide used by moderators is provided in Appendix A. Recruitment and data collection continued until data saturation was reached, defined as the point at which additional FGs no longer generated substantially new insights or themes [28]. This approach ensured the inclusion of participants whose experiences were particularly informative for capturing the complexities of interprofessional collaboration in primary care.

### 2.4. Data Analysis

All anonymized FG transcripts were analyzed using RTA, following Braun and Clarke’s six-phase framework: (a) familiarization with the data; (b) generation of initial codes; (c) identification of preliminary themes; (d) review and refinement of themes; (e) definition and naming of themes; and (f) production of the final analytical narrative [27,28]. RTA was chosen for its theoretical flexibility, capacity to capture both semantic and latent patterns of meaning, and emphasis on the interpretative and reflexive role of the researcher within a constructivist epistemological stance [28,35].

Data analysis was conducted concurrently with data collection, following an iterative and reflexive process that allowed for insights from earlier FGs to inform subsequent sessions and probing strategies [27]. This dynamic approach facilitated ongoing refinement of codes and themes and ensured that the evolving analysis remained closely aligned with participants’ lived experiences. Two researchers (both MSc-level nurses with advanced training in qualitative inquiry and Reflexive Thematic Analysis) independently conducted line-by-line coding of the transcripts, assigning concise labels to discrete units of meaning [4]. Throughout this process, descriptive, linguistic, and conceptual notes were generated, alongside reflexive memos derived from field notes and observations made during the FGs [6,7]. This memoing supported deeper engagement with the data, enabling the identification of subthemes that captured the nuanced dimensions of interprofessional collaboration and the complexities of participant interactions.

Codes were synthesized and organized into preliminary themes, which were iteratively reviewed and refined through collaborative discussion to ensure they accurately reflected recurrent patterns and shared meanings across participants’ narratives [5,9]. Subthemes were then grouped into broader thematic categories aligned with the study’s research objectives. Discrepancies in coding or theme development were resolved through reflective dialogue among the primary analysts, with senior researchers consulted as needed to ensure methodological rigor, coherence, and interpretive depth. Representative quotations were selected to illustrate and substantiate each theme within the final narrative.

Consistent with the epistemology of RTA, the analysis prioritized interpretative depth over quantification; frequency counts and inter-coder reliability metrics were not reported, as they are not aligned with the method’s principles [27,28]. The process was inherently iterative, collaborative, and reflexive, producing a rich, contextually grounded, and nuanced understanding of the patterns and meanings embedded in participants’ accounts [35].

### 2.5. Rigour and Reflexivity

To ensure methodological rigor and enhance trustworthiness, the study adhered to established criteria encompassing credibility, transferability, and dependability [36]. Several strategies were employed to strengthen the accuracy and integrity of both data collection and analysis. First, all FG transcripts were fully transcribed verbatim, ensuring transparency and consistency throughout the analytic process [36]. Second, researchers applied the bracketing technique, documenting pre-existing assumptions related to the research topic to mitigate potential biases and enhance the validity of findings [30]. Third, an independent analyst (PhD in Nursing Science) with extensive qualitative research experience, who was not involved in the original data collection, conducted an initial coding of the transcripts. Subsequently, a qualitative research expert (MSc-level Nurse) with experience in phenomenological methods reviewed and validated both the coding process and thematic organization, ensuring rigor and credibility. This independent coding was subsequently compared with that of the primary analysts, facilitating triangulation of perspectives and enriching the development of the thematic framework.

Following this, a qualitative research expert with experience in phenomenological methods reviewed and validated both the coding process and thematic organization. Emergent themes were then subjected to collective team discussion to achieve consensus and a shared understanding of their meaning. In cases of disagreement, the research team revisited relevant data excerpts to refine interpretations. Divergent perspectives on theme identification were resolved through collaborative dialogue, ensuring that all interpretations were grounded in the data.

Finally, member checking was conducted with a purposive subsample of three participants who had agreed to further contact. They were purposefully selected to ensure representation of both professional roles (one GP and two FCNs) and of different regional contexts, reflecting the heterogeneity of the study sample. These participants received summaries of preliminary themes via email and were invited to provide feedback on the clarity, relevance, and resonance of the interpretations. Their input confirmed the thematic structure, with no substantial amendments required, thereby reinforcing the credibility of the study [37].

### 2.6. Ethical Considerations

Ethical approval for this study was granted by the Research Ethics Board of University of Parma (Protocol No. 0266537—21 October 2022). Prior to participation, all individuals provided informed consent and were assured of confidentiality, anonymity, and the voluntary nature of their involvement. Data collection, management, and storage complied with international ethical standards and the General Data Protection Regulation (GDPR). Audio recordings and transcripts were anonymized and securely stored on encrypted institutional servers accessible only to authorized research team members. The study protocol was carefully designed to minimize potential risks and to uphold participant autonomy and respect throughout all stages of the research process.

## 3. Results

A total of four FGs were conducted involving 21 participants: 8 GPs and 13 FCNs. The sample comprised 14 women and 7 men, with a mean age of 41 years (range: 27–60). Participants were recruited from three Italian regions—Tuscany (*n* = 6), Lombardy (n = 12), and Emilia-Romagna (*n* = 3)—and were employed across diverse primary care settings, including group medical practices, CdC, and outpatient clinics. All participants had a minimum of one year of professional experience in primary healthcare, with individual experience ranging from 1 to 23 years. It should be noted that the ‘Years of Collaboration’ column in Table 1 reflects participants’ total interprofessional experience in primary care, which may exceed the duration of the current structured GP-FCN program. Two participants had recently joined the program and had not yet completed a full year of collaboration at the time of data collection, but they were included as they could provide meaningful insights based on early-stage collaborative experiences. Sociodemographic and professional characteristics of the participants are detailed in Table 1.

Analysis of the four FGs yielded four main themes and sixteen subthemes, which are presented below along with illustrative quotations. For a concise overview of the qualitative findings, Appendix B summarizes the main themes, subthemes, and selected quotations that exemplify participants’ perspectives on key dimensions of interprofessional collaboration between GPs and FCNs. Thematic analysis did not reveal major differences across the three regional contexts. Participants from all areas described similar dynamics, challenges, and facilitators in interprofessional collaboration, suggesting that these experiences are shared across organizational and territorial settings.

### 3.1. Theme 1: Barriers to Effective Interprofessional Practice

Interviews highlighted multiple obstacles that impede cohesive teamwork and productive collaboration between GPs and FCNs. Four subthemes emerged, reflecting structural, relational, and perceptual challenges.

#### 3.1.1. Lack of Knowledge and Role Recognition

A central barrier was the insufficient understanding of FCNs’ competencies and responsibilities among GPs, especially among older practitioners. This gap often fostered mistrust or skepticism, complicating role clarity. The evolution of FCNs’ responsibilities further blurred boundaries, increasing confusion: “There is still a bit of fear or difficulty in defining the boundaries, therefore in defining the role, that is, where the doctor intervenes, where and how the nurse intervenes, and there is still a sort of ambivalence” (FG1_sg3). GPs were sometimes hesitant about the practical utility of FCNs, limiting openness to collaboration. Nurses expressed frustration about being perceived merely as support staff, constrained by hierarchical dynamics: “Perhaps even a sort of hierarchy is still present in the minds of some, and this becomes a huge limitation” (FG2_sg3).

#### 3.1.2. Communication and Relational Difficulties

Both physicians and nurses highlighted the presence of communication and relational difficulties that negatively affected collaboration: “We do not have a shared platform to upload information, and this creates a problem.” (FG1_sg2). One of the main barriers identified by professionals was the lack of common management systems, which led them to rely on telecommunication tools and informal channels—such as WhatsApp, email, and phone calls—to exchange information and keep each other updated on their work: “There are nurses I don’t even know—I mean, I speak with them on the phone or via WhatsApp and so on, but I don’t know who they are; I have never met them.” (FG1_sg3); and “the second issue, which may seem trivial but has emerged repeatedly, is the impossibility of sharing information… and often in completely inappropriate ways, like voice messages on WhatsApp. In practice, we end up exchanging observations with the family nurse via WhatsApp, because we lack a shared platform for entering information, and this creates a real problem.” (FG1_sg2). Many participants reported that communication often took place through non-integrated portals. Additional barriers included difficulties in reaching colleagues and the absence of a shared physical workspace, which further hindered communication and teamwork.

Relational challenges also emerged, stemming from individual personality traits that complicated the mutual understanding necessary for effective collaboration. According to some professionals, this resulted in a certain “*neglect*” of relationship-building (FG4_sg1), a lack of openness to change, and a prevailing sense of “*frustration*” (FG4_sg2).

#### 3.1.3. Insufficient Time for Relationship Development

Professionals repeatedly emphasized the lack of time available to invest in building relationships. “It is very difficult, because they have so many things to do, so it is hard to find the time to talk […] with them.” (FG3_sg6); and “The biggest obstacle I experience […] is the shortage of nursing staff, because today we feel it very strongly when trying to structure work with us that goes beyond direct care.” (FG1_sg4). A heavy workload and staff shortages, reported across many settings, were among the most frequently cited reasons for the lack of time—both for interpersonal interactions and for collaborative work: “The biggest obstacle is precisely that we manage to meet very rarely. I mean, almost never… and in my opinion, that is a major barrier.” (FG3_sg6). FCNs and GPs expressed not only the difficulty of finding time to get to know one another, but also the challenge of dedicating time to case discussions and the joint planning of shared activities. The absence of time explicitly devoted to nurturing the relationship itself—acknowledged as necessary to “learn how to work in a team, because it cannot be improvised” (FG2_sg3)—was perceived as a significant barrier to clinical collaboration.

#### 3.1.4. Structural and Organizational Misalignment

Systemic differences between FCNs and GPs created barriers to coordination. FCNs are institutionally employed, whereas GPs often work as independent contractors, resulting in inconsistent workflows and objectives: “They want to collaborate with us but must meet company commitments that do not always align with our expectations” (FG4_sg2). Geographic assignment of FCNs versus patient choice of GPs further complicated collaboration: “If we had had an individual relationship, the transition would probably have been quicker” (FG1_sg4).

### 3.2. Theme 2: Facilitators of Productive Interprofessional Collaboration

Despite these obstacles, several enabling factors emerged that fostered effective teamwork, following the five subthemes below.

#### 3.2.1. Willingness to Collaborate and Generational Openness

Participants highlighted the importance of openness to collaboration, emphasizing the need to engage with new professional roles and embrace change. Younger generations of GPs were described as particularly receptive to teamwork and appreciative of the contributions that nurses bring to patient care: “There are some young doctors… who are more open to dialogue… They looked favourably on us because they were no longer alone” (FG3_sg6). Generational openness was associated with relational engagement, adaptability, and emotional investment, which enhanced team functioning and professional fulfillment.

#### 3.2.2. Recognition of Roles and Expertise

Mutual understanding and respect of professional roles emerged as key facilitators. FCNs highlighted the importance of recognition for their evolving, holistic contributions: “Recognition of the effort we are making, of our professionalism, of our great change” (FG2_sg1). “For me, the doctor must be a resource as I must be a resource for the doctor; there is no question of hierarchy” (FG2_sg5) stated a participant. Shared physical space, structured meetings, and informal interactions (e.g., coffee breaks) strengthened communication and trust: “Having had the opportunity to get to know each other and have coffee together, in my opinion, in the initial phase was fundamental” (FG4_sg1).

#### 3.2.3. Shared Goals

A shared focus on patient-centered goals was described as the cornerstone of successful collaboration. Alignment around patient care enhanced trust and promoted proactive teamwork: “When the patient is central and we both share our positions, the relationship works” (FG2_sg3). Maintaining awareness of this shared goal, despite individual differences and occasional rigidity, was considered critical to achieving collaborative success: “Knowing that you are looking for the same thing increases trust in the other and, therefore, collaboration” (FG1_sg3).

#### 3.2.4. Shared Space and Time

The sharing of physical space and time emerged as a critical facilitator of collaboration. Co-location within the same healthcare setting fostered dialogue and strengthened professional relationships: “Living in the same facility greatly facilitated team work with the nurses” (FG1_sg4). Both structured team meetings and informal interactions, such as coffee breaks, were considered essential for cultivating trust and mutual understanding: “Having the opportunity to get to know each other, to have coffee together, in my opinion, was fundamental during the initial phase” (FG4_sg1).

#### 3.2.5. Communication and Interpersonal Skills

Effective collaboration was built on mutual trust, active listening, patience, and empathy—core elements consistently highlighted by participants as essential for productive teamwork. These interpersonal skills allowed for professionals to navigate complexity, manage conflicts, and respond effectively to evolving care needs. As one participant emphasized, “Good communication skills… how we communicate with each other is very important” (FG3_sg1), underscoring that relational competence, alongside technical coordination, plays a critical role in shaping team dynamics. Participants also stressed the significance of emotional attunement and responsiveness, particularly in challenging or ambiguous situations. Complementing these relational dimensions, creativity emerged as a key facilitator of collaboration, especially in devising adaptive strategies and addressing context-specific challenges. As one participant illustrated, “Trying to bring out who does what and how we do it, even in a creative way” (FG4_sg1), demonstrating how imaginative thinking and collaborative problem-solving enable professionals to transcend rigid role boundaries and co-construct flexible approaches to care.

### 3.3. Theme 3: Team Formation Process

Team-building was described as a non-linear, evolving process, characterized by learning, trust formation, and shared professional development.

#### 3.3.1. Phases of Team Development

Initial phases were defined by mutual observation, cautious engagement, and the gradual construction of trust. Participants emphasized the importance of having sufficient time and space to build interpersonal familiarity and collaboratively define working methods. One participant noted, “Also allow them to get to know us, because only in this way… can we break down resistance and mistrust” (FG3_sg5). Collaborative activities such as joint clinical decisions, home visits, and interdisciplinary meetings were reported to accelerate cohesion: “We constantly visit together; they help us and we help them… a great opportunity for growth for everyone” (FG3_sg2).

#### 3.3.2. Shared Professional Values

Team identity was shaped by values such as mutual support, reciprocity, and a sense of belonging, collectively forming the ethical foundation of interprofessional collaboration. Participants consistently emphasized that perceiving oneself as part of a cooperative system was not only professionally enriching, but also indispensable to the quality of care delivered. As one participant expressed, “Each piece supports the other… to do the best job possible” (FG1_sg1), illustrating how interdependence and shared responsibility strengthened both performance and morale. Another participant noted, “What do we do? What can I do? And from there, various doors open” (FG2_sg1), highlighting how collaborative values encouraged proactive engagement and creative thinking. These values provided a relational framework extending beyond daily tasks, influencing attitudes toward teamwork, conflict resolution, and collective accountability.

#### 3.3.3. Problem-Solving Strategies

Professionals highlighted reflective practices as essential tools for navigating fatigue, interpersonal misunderstandings, and the inherent challenges of collaborative work. Reflection was not considered passive, but rather an active and intentional practice of stepping back, reassessing, and recalibrating individual and team responses. One participant noted, “The attitude to reflect and say: ‘What is going wrong?’ … proceed, otherwise you don’t move forward” (FG4_sg1), underscoring the link between reflection, resilience, and forward momentum. Another described how empathy and contextual sensitivity helped identify underlying challenges: “Maybe it could be the difficulty of not being able to manage the complexity of a case at home, and so when you realize that this is the difficulty, you try to answer, perhaps concerning activating a service or a consultant” (FG1_sg3). Open dialogue and the ability to reframe complex situations were considered vital to maintaining cohesion and avoiding stagnation.

#### 3.3.4. Specific Training Needs

Participants called for more focused training in relational skills, interdisciplinary practices, and community-based care. Shared learning experiences were viewed as opportunities to build common ground and align methodologies. One participant stated, “Experience is needed because what is done in the hospital is completely reversed compared to what is done at home” (FG3_sg1). The need for shared interprofessional training—particularly in chronic care, palliative care, and community health—was widely expressed: “Starting from scratch, all together… would help the relationship between family doctors and nurses a lot” (FG1_sg1).

### 3.4. Theme 4: Transformation of Work Practices

The final theme reflects tangible shifts in clinical practice, professional satisfaction, and health outcomes resulting from interprofessional collaboration. These changes were explored across three subthemes: health outcomes, work methodologies, and transformative dynamics.

#### 3.4.1. Health Outcomes

Participants reported improved outcomes not only for patients, but also for professional well-being. Team-based care was associated with greater empathy, job satisfaction, and enhanced capacity to manage complex cases collaboratively: “You can work in a team, with empathy, and find satisfaction for everyone… truly a great satisfaction” (FG3_sg2). Collaborative models allowed for comprehensive case management and increased personalization of care, reinforcing human connection and trust with patients. As one participant affirmed, “We are finally able to take charge of previously unthinkable cases… through a network action that was previously unimaginable” (FG4_sg1).

#### 3.4.2. Work Methodologies

Care planning evolved into a more shared, iterative, and responsive process, reflecting the dynamic nature of interprofessional collaboration in primary care. Rather than following predefined protocols, professionals engaged in continuous dialogue to co-design individualized care pathways and distribute responsibilities in real time according to patients’ changing needs. One participant emphasized, “Together, we decide on the path to take… we make decisions together. This is very, very nice” (FG2_sg2), highlighting the shift from unilateral decision-making to collective ownership of care. This collaborative ethos promoted coordinated action and mutual accountability, as each professional contributed distinct yet complementary expertise. Moreover, interprofessional collaboration encouraged a pragmatic and flexible approach to professional roles: “Crossing the border can be convenient… but remaining flexible is fundamental” (FG1_sg1), reflecting a willingness to move beyond rigid role definitions when necessary to ensure continuity and comprehensiveness of care.

#### 3.4.3. Transformative Dynamics

Professional practices transitioned from isolated, profession-specific performances to dynamic, co-created care strategies grounded in interdependence and a shared vision. This transformation was driven by mechanisms such as clear team reference points, strategic role distribution, and integration of local, community-based knowledge into care planning. One participant observed, “Let’s start doing things differently… creating alliances… this is what I expect from having community nurses” (FG4_sg1), highlighting a shift toward proactive, partnership-based care. Participants emphasized that shared understanding of local networks, services, and resources was crucial for achieving comprehensive, patient-centered outcomes: “Having a good knowledge of the network… can make a difference on the patient” (FG3_sg5). This reflects the awareness that collaboration extends beyond the clinical dyad to include social, institutional, and territorial actors.

## 4. Discussion

Our study provides an in-depth qualitative exploration of interprofessional collaboration between GPs and FCNs in Italian primary healthcare using RTA. By examining the experiences of 21 professionals across three regions, this research illuminates the often-overlooked relational and organizational dynamics that shape teamwork in real-world primary care settings. The study’s central thesis is that effective interprofessional collaboration is less a product of formal protocols and more a construct of trust, shared identity, and iterative co-learning. This perspective challenges conventional models of integration that focus predominantly on structural or procedural alignment, highlighting the transformative potential of relational and experiential processes. The innovative contribution of this study lies in its detailed articulation of how team-building, role recognition, and professional identity function as both enablers and barriers to collaboration, providing actionable insights for workforce development, organizational design, and policy in community-based care [4,5,38].

Our findings highlight persistent obstacles to effective GP–FCN collaboration, including unclear role delineation, hierarchical perceptions, generational differences, and systemic misalignments [39,40,41,42]. These barriers reflect both relational and structural challenges. Despite the recognized potential of collaborative practice, participants reported that professional silos and fragmented organizational cultures often hindered seamless teamwork, underscoring the need for targeted interventions. Ambiguities in FCN competencies, especially among GPs trained under traditional physician-centered models, were frequently cited, while nurses reported frustration at hierarchical perceptions limiting recognition of their contributions. These insights confirm previous evidence on the challenges of interprofessional collaboration in PHC [39,40].

Conversely, key enablers of collaboration emerged, notably trust, mutual respect, shared professional identity, and informal relational interactions such as co-location and joint clinical activities [39,43,44,45]. These elements fostered psychological safety and iterative learning, supporting the development of effective teamwork. Regular team meetings, shared documentation, and open communication were also cited as practical strategies to enhance collaborative practice. Participants emphasized that relational factors often outweighed structural alignment, reflecting the transformative potential of interpersonal processes in shaping coordinated, patient-centered care.

Participants emphasized the importance of interprofessional training in clarifying roles, enhancing professional identity, and promoting teamwork skills [43,46]. Exposure to integrated care models and interprofessional education contributed to greater openness among younger GPs and reinforced the recognition of FCN competencies. These insights suggest that structured training and reflective learning opportunities are pivotal to sustaining high-functioning interprofessional teams in PHC. Team-building processes were reported to act as dynamic mechanisms allowing for professionals to negotiate roles, learn collaboratively, and adapt practices according to patient and contextual needs.

Finally, collaborative dynamics were linked to tangible changes in practice, including improved care coordination, patient-centered approaches, and adaptive responses to complex cases [39,41,42,45]. Team-building enabled the evolution of roles according to patient needs and contextual demands, demonstrating that interprofessional collaboration is a dynamic, co-constructed process rather than a static protocol. Embedding these practices into organizational structures, supported by leadership and policy alignment, is essential for long-term sustainability and the enhancement of population health outcomes. Participants highlighted that without relational space, psychological safety, and iterative learning opportunities, procedural integration alone was insufficient to achieve genuine transformation in practice.

### 4.1. Limitations

This study presents several limitations that must be acknowledged. First, it relied on a purposive sample of GPs and FCNs from three Italian regions. While providing contextual diversity, the relatively small sample may not fully capture the heterogeneity of interprofessional dynamics across the national primary care system, limiting transferability [47]. Second, the use of FGs, although effective for eliciting rich narratives, may have constrained dissenting opinions due to existing professional hierarchies or perceived power differentials [48]. Third, conducting all sessions virtually facilitated participation, but potentially reduced non-verbal cues and spontaneous interactions, highlighting the need for enhanced facilitation in digital formats [49]. Finally, the cross-sectional design provides only a snapshot of collaborative dynamics, without capturing their evolution over time; longitudinal studies could illuminate how interprofessional relationships develop, adapt, and influence patient and system-level outcomes [50].

### 4.2. Implications for Practice

The findings provide actionable insights for strengthening interprofessional collaboration in primary care. Structured opportunities for relationship-building, including co-location, joint decision-making, and informal interactions, were identified as critical enablers of trust and team cohesion [51,52]. Role recognition and mutual respect emerged as central to effective collaboration, highlighting the value of interprofessional training that emphasizes team dynamics, professional identity, and scope of practice, in line with WHO recommendations for Interprofessional Education [43,46]. Furthermore, organizational alignment—including contractual, digital, and policy support—alongside facilitative leadership, is necessary to sustain collaboration and nurture collective accountability [40,41,42].

### 4.3. Future Perspectives

Future efforts should focus on consolidating the integration of FCNs in primary care, enhancing both visibility and legitimacy of their role. Longitudinal research is needed to examine how interprofessional collaboration evolves across territorial care models and impacts care continuity, patient satisfaction, and population health outcomes [53,54]. Interprofessional training tailored to the Italian context, where public nurses and self-employed GPs coexist, could provide evidence on strategies that foster resilient collaboration [40,55]. Policymakers might consider local innovation hubs or learning communities to co-develop and test collaborative practices, embedding interprofessional work into the system and promoting patient-centered, integrated primary care [41,42].

## 5. Conclusions

This study provides an in-depth and empirically grounded understanding of the processes, barriers, and facilitators shaping collaboration between FCNs and GPs in Italian primary care. The findings demonstrate that interprofessional collaboration is not achieved merely through structural proximity or top-down directives, but rather evolves progressively through shared experiences, reciprocal recognition, and the co-construction of trust. Specifically, Theme 1 identifies key barriers, such as limited role recognition, communication difficulties, insufficient time, and structural misalignment, while Theme 2 highlights facilitators, including willingness to collaborate, recognition of roles and expertise, shared goals, shared space and time, and interpersonal skills. This explicit distinction clarifies the dual nature of factors influencing collaboration and directly aligns with the study’s objectives. Within this dynamic, team-building emerged as a central transformative mechanism, while professional identity and role clarity functioned simultaneously as potential obstacles and critical enablers of collaborative practice.

The practical implications of these findings are significant: fostering authentic interprofessional collaboration requires strategic investment in relational, educational, and policy infrastructures that actively support teamwork. These investments can enhance professional well-being, strengthen team cohesion, and improve the adaptability, equity, and patient-centeredness of care delivery. By highlighting the relational and experiential foundations of collaboration, this study contributes to the evidence base guiding the design of sustainable, integrated primary healthcare systems and emphasizes the need to embed interprofessional practices as a core element of healthcare transformation.

## Figures and Tables

**Table 1:** Socio-demographic and professional characteristics of the sample.

Code	Age	Sex	Role	City	Professional Training	Work Setting	Y/Experience	Y/Current Role	Y/Collaboration
FG1_sg1	38	F	GPs	Barberino di Mugello, Florence, Tuscany	General Medicine	Group Practice	12	6	12
FG1_sg2	42	F	GPs	Florence, Tuscany (urban outskirts)	Specific Training Course in General Medicine	Group Practice	12	5	3
FG1_sg3	41	F	GPs	Florence, Tuscany	Degree in Medicine and Surgery—Specific Training Diploma in General Medicine	Association Practice	12	4	2
FG1_sg4	44	F	GPs	Florence, Tuscany (urban outskirts)	Specific Training Course in General Medicine	Community Health Center	18	4	3
FG2_sg1	53	F	FCNs	Brianza, Lombardy	1st Level Master’s in Case Care Management in Hospital and Community	Community Health Center, Giussano	32	2	2
FG2_sg2	27	M	FCNs	Milan, Lombardy	Nursing Degree, in progress: 1st Level Master in Family and Community Nursing	Home Care, Milan Martesana	6	2	4
FG2_sg3	48	F	FCNs	Crema, Lombardy	Nursing Degree, in progress: 1st Level Master in Family and Community Nursing	Territorial Hub, ASST Crema	28	1	1
FG2_sg4	34	F	FCNs	Tradate, Varese, Lombardy	Bachelor of Nursing	Community Health Center, Tradate	10	1	1
FG2_sg5	29	F	FCNs	Pavia, Lombardy	Nursing Degree, 1st Level Master in Family and Community Nursing	Outpatient Nurse, ASST Pavia	5	5	1
FG2_sg6	NA	M	FCNs	NA	Nursing Degree, 1st Level Master in Family and Community Nursing	NA	27	8	NA
FG3_sg1	50	F	FCNs	Florence, Tuscany	Regional Nursing Diploma	Health Center Le Piagge, Florence	28	23	23
FG3_sg2	47	F	FCNs	Florence, Tuscany	Bachelor of Nursing	District Health Center Le Piagge, Florence	23	10	10
FG3_sg3	47	F	FCNs	Ferrara, Emilia Romagna	MSc in Nursing Science	Health Center, Ferrara	26	NA	2
FG3_sg4	34	F	FCNs	Crema, Lombardy	Nursing Degree, 1st Level Master in Family and Community Nursing	Community Health Center, ASST Crema	11	2	2
FG3_sg5	37	F	FCNs	Crema, Lombardy	Nursing Degree, 1st Level Master in Family and Community Nursing	Territorial FCN Ambulatory, ASST Crema	14	4	4
FG3_sg6	33	M	FCNs	Tradate, Varese, Lombardy	Nursing Degree, 1st Level Master in Family and Community Nursing	Community Health Center, Tradate	9	1	1
FG3_sg7	30	M	FCNs	Milan, Lombardy	Nursing Degree, in progress: 1st Level Master in Community Nursing	Nemo Clinical Center, Niguarda Hospital, Milan	NA	1	1
FG4_sg1	42	M	GPs	Ferrara, Emilia Romagna	Specific Training Course in General Medicine	Group Practice	5	5	1
FG4_sg2	39	F	GPs	Ferrara, Emilia Romagna	General Practitioner	Group Practice	8	1,5	1
FG4_sg3	NA	M	GPs	Rivolta d’Adda, Cremona, Lombardy	General Practitioner	Group Practice	30	30	0
FG4_sg4	NA	M	GPs	Rivolta d’Adda, Cremona, Lombardy	General Practitioner	Group Practice	30	30	0

Note: “Years of Collaboration” reflects total professional interprofessional experience in primary care. Some participants’ years of collaboration may exceed the duration of the current GP-FCN program. Two participants had less than one year in the current program, but were included due to their early-stage collaborative insights.

## Data Availability

The datasets generated and analyzed during the current study are not publicly available due to privacy and confidentiality agreements with participants, but are available from the corresponding author on reasonable request.

## Appendix A. Focus Group Guide for GPs and FCNs

Purpose:
  The focus group guide was designed to facilitate discussion on interprofessional collaboration between General Practitioners (GPs) and Family and Community Nurses (FCNs). The aim was to elicit participants’ experiences, perceptions, expectations, and suggestions regarding collaborative practice in primary care.
Moderator Instructions:
  1. Begin by welcoming participants and explaining the purpose of the session.
  2. Emphasize confidentiality and the voluntary nature of participation.
  3. Encourage open discussion and assure participants that there are no right or wrong answers.
  4. Maintain a neutral stance; use active listening techniques (paraphrasing, summarizing, interpretive questioning) to clarify meanings and encourage reflection.
  5. Observe group dynamics and take field notes on non-verbal cues, interactions, and contextual factors.
Session Structure:
  - Introduction (5–10 min):
    ○ Welcome participants and introduce the study aims.
    ○ Explain session format and guidelines for discussion.
    ○ Ensure informed consent is obtained.
  - Opening Question:
    ○ “How would you describe the relationship between FCNs and GPs?”
    ○ Purpose: To elicit broad narratives and allow for participants to frame their own experiences.
  - Core Discussion Questions:
    ○ “In your opinion, what are the necessary attitudes for teamwork between you and GP/FCN?”
    ○ “What are the critical aspects you experience regarding collaboration between GP and FCN?”
    ○ “What do you expect from the relationship between you and the other professional?”
    ○ “What do you think the other expects from you?”
    ○ “What is needed for effective collaboration?”
Probing Prompts:
  - “Can you provide an example?”
  - “Can you elaborate on that?”
  - “How did that situation make you feel?”
  - “Why do you think that occurred?”
Closing (5–10 min):
  - Summarize main points discussed.
  - Ask participants if they would like to add anything further.
  - Thank participants for their time and contributions.
Notes for Moderators:
  - Encourage all participants to contribute equally.
  - Adapt the order and wording of questions flexibly according to the discussion flow.
  - Take detailed field notes on non-verbal behavior, interactions, and contextual factors.

Ensure the session remains focused on experiences, perceptions, and collaboration dynamic

## Appendix B. Theme and Subthemes Resulted

**Themes**	**Subtheme**	**Quotations**
Barriers to Effective Interprofessional Practice	Lack of knowledge and role recognition	“There is still a bit of fear or difficulty in defining the boundaries, therefore in defining the role, that is, where the doctor intervenes, where and how the nurse intervenes, and there is still a sort of ambivalence.” FG1_sg3 “Perhaps even a sort of hierarchy is still present in the minds of some, and this becomes a huge limitation.” FG2_sg3
	Communication and relational difficulties	“We lack a shared platform for entering information, which creates a problem.” FG1_sg2 “There are nurses that I don’t even know, that is, I talk to them on the phone and via… via WhatsApp, etc., but I don’t know who they are, I’ve never met them.” FG1_sg3 “… instead we use very rudimentary tools that…that we can’t perfect and can’t lead, to improve to say now that as a company we use the regional folder, after a year that we’ve been working together, someone could have said… but let’s develop a link for them, right? That’s frustrating, but it’s fundamental because it actually gives you an extra edge, also on many other projects that don’t only concern misfortunes, because the complex cases are our misfortunes, but the patients who are fortunate are the majority” FG4_sg1
	Insufficient time for relationship development	“It’s a lot of work because they have so many things to do, so it’s hard to find the time to talk […] with them.” FG3_sg6 “The biggest obstacle I experience […] is the lack of nursing staff, because today we feel it a lot, in structuring a job with us that goes beyond assistance…” FG1_sg4 “Four words: skills, communication, learning to work in team working because you can’t improvise.” FG2_sg3
	Structural and Organizational Misalignment	“They want to collaborate with us, but they must meet company commitments that do not always align with our expectations.” FG4_sg2 “If we had had an individual relationship, the transition would probably have been quicker”. FG1_sg4 “We come up against the fact that they too struggle to make themselves completely available and completely autonomous because they answer to the company” FG4_sg2
Facilitators of productive interprofessional collaboration	Willingness to collaborate and generational openness	“There are some young doctors… it is not a valid example… who are… more open to dialogue and therefore also…. They looked favorably on us when we arrived because at least they were no longer alone in managing certain things, and they knew that if they called us, we would be there for the patients…” FG3_sg6 “If every now and then we do things that they should do, I mean no…no…nothing happens, you know, if we meet them halfway, in this sense here, that is, if sometimes it happens that the family nurse can ask us or we see that he is in difficulty if we can do something, we do it in short, nothing happens.” FG1_sg2 “… a job… That is never perfect […] but it is the best possible” FG1_sg1 “Make a big effort to remove hierarchies and to truly put everyone on the same level” FG1_sg4 “If now and then we do things that they should do, I mean nothing happens, you know, if we meet them halfway” FG1_sg2
	Recognition of roles and expertise	“What do I expect from the relationship with the GP? Recognition. Recognition of our effort, our professionalism, and the great change we are making, because for us it is an epochal change, there is a mental, but also a physical change.” FG2_sg1 “For me, the doctor must be a resource as I must be a resource for the doctor, there is no question of hierarchy.” FG2_sg5
	Shared goals	“I believe I can say that when the collaboration with the doctor meets certain criteria for goodness, it is when the patient becomes central for both. When the patient is central and we both share our positions, the relationship works, the objectives are aligned, and even if there are difficulties, it can be done.” FG2_sg3 “Always keeping the objective in mind, even overcoming some rigidities […] is an indispensable attitude […] it is not easy, but I believe that it has often been the key to success.” FG1_sg2 “… knowing that you are looking for the same thing” FG1_sg3 “The location is fundamental, if they worked here, dialogue and collaboration would inevitably be created, good relationships would be established.” FG2_sg4 “…living in the same house has greatly facilitated teamwork with nurses” FG1_sg4 “We share the same physical location as the family and community nurse, who lives right next door, so I wrote words of sharing, support, and team.” FG1_sg4 “Having had the opportunity to get to know each other and have coffee together, in my opinion, in the initial phase was fundamental.” FG4_sg1
	Shared space and time	“Having the opportunity to get to know each other, to have coffee together, in my opinion, was fundamental during the initial phase” (FG4_sg1). “Living in the same facility greatly facilitated team work with the nurses” (FG1_sg4)
	Communication and interpersonal skills	“Good communication skills…and…therefore…how we communicate with each other is very important…” FG3_sg1 “Patience in terms of […] listening. I take a step back, I make this reflection, thinking above all about the most difficult situations to manage. So having […] the patience to listen to understand the difficulties the person on the other end is experiencing”. FG2_sg3 “Trying to bring out who does what and how we do it, even in a creative way” FG4_sg1 “Personally, the most important tool is Google Meet; without it, we would not make any progress, as that is where we hold meetings.”. FG4_sg1
Team formation process	Phases of team development	“Also allow them to get to know us, because only in this way, only by letting us know each other, can we break down resistance and mistrust”. FG3_sg5 “When you try to tiptoe in […] “Look, I saw this thing and I wanted to share it with you […] what if we do it like this?” Make them feel part of the decision anyway”. FG3_sg6 “We constantly visit together; they help us and we help them. If there is a problem, and we see that a situation does not make sense to us, we discuss it and assess it together. It is truly precious and a great opportunity for growth for everyone.” FG3_sg2
	Shared professional values	“In this relationship in which each person brings their piece, each piece supports the other in holding together to do the best job possible…”. FG1_sg1 “What do we do? What can I do?» And from there, various doors open”. FG2_sg1 “Indispensable because since I closely collaborate with the FCNs I can no longer do without it”. FG1_sg1
	Problem-solving strategies	“The attitude to reflect and say: «Ok, what is going wrong? We are arguing…» or «We are tired». This means that this misfortune must teach us something [and] proceed, otherwise you end up arguing and then you don’t move forward.” FG4_sg1 “So, having the patience to listen and try to understand the difficulties the person on the other side is experiencing. Maybe it could be the difficulty of not being able to manage the complexity of a case at home, and so when you realize that this is the difficulty, you try to answer, perhaps concerning activating a service or a consultant”. FG1_sg3
	Specific training needs	“Experience is needed because what is done in the hospital is completely reversed compared to what is done at home … because it still enters people’s homes”. FG3_SG1 “At that point, starting from scratch, all together, the difference between what I have to do and what you have to do is flattened: we started the path together. […] utopia, fantasies… but it would be useful. […] and it would help the relationship between family doctors and nurses a lot… there would be the family team, that is, the group of health workers and others who take care of that person”. FG1_sg1
Transformation of work practices	Health outcomes	“You can work in a team, with empathy, and find satisfaction [for] everyone: the patients, the doctor, and us. It is a good way of working together. When you manage to do it, [it is] truly a great satisfaction”. FG3_sg2 “Extraordinary things come out, that is, we are finally able to take charge of previously unthinkable cases, stabilize them, and manage them most appropriately. Here we have a series of patients […] that we are now able to manage through a network action that was previously unthinkable and not even imaginable”. FG4_sg1 “It is the team and the work together that relieves you of the responsibility of having a person in charge…so the weight you feel on your shoulders changes a lot when there is real work…when there is integrated work in a team where it is not only an exchange of performances but a transformation [towards] a decision that is shared on the person”. FG1_sg4
	Work methodologies	“Together, we decide on the path to take and we collaborate, in the sense that we are a small team of two people: he on medicine and I on nursing, and we also collaborate on diagnostic pathways at times, and we make decisions together. This is very, very nice”. FG2_sg2 “… a whole series of problems; therefore not only medical problems but also social care problems”. FG4_sg2 “We have coined the word fantasy within the relationship: what is needed for a collaboration with the doctor, but also with the citizen? Because in the face of equal needs with different people, the solution is sometimes imaginative. Because in the territory, what you find is that there is no path that is always the same for everything; therefore, on the contrary, it is always very personalized. So even the relationship that exists with the doctor and the nurse and the situation you are in charge of becomes very difficult if you do not have a bit of imagination”. FG2_sg1 “… we get to know each other in this protected perimeter” FG4_sg1 “Here, in my opinion, that was important because from there you can now move on to other projects.” FG4_sg1
	Transformative dynamics	“Start making plans and that are not only as I said before are on the disease, on bad luck, on the complex case but that also concern axes such as prevention up to the promotion of health […]… let’s start doing things a little different: planning, knowing the territory, investigating it, seeing who are the actors that we can co-opt, creating alliances… in short this is what I expect from having community nurses”. FG4_sg1 “Having a good knowledge of the network…[…] to understand what beyond our roles […] can make a difference on the patient… [can] help both to activate further actors who can, for their little piece, enter the path and make the way to complete and… respond to the patient’s need”. FG3_sg5
